# The Potential Value of Sleep Hygiene for a Healthy Pregnancy: A Brief Review

**DOI:** 10.1155/2014/928293

**Published:** 2014-02-17

**Authors:** Zachary M. Ferraro, Jean-Philippe Chaput, Andrée Gruslin, Kristi B. Adamo

**Affiliations:** ^1^Children's Hospital of Eastern Ontario Research Institute, Healthy Active Living and Obesity (HALO) Research Group, 401 Smyth Road, Ottawa, ON, Canada K1H 8L1; ^2^School of Human Kinetics, Faculty of Health Sciences, University of Ottawa, Ottawa, ON, Canada K7N 6N5; ^3^The Ottawa Hospital Research Institute, 501 Smyth Road, Ottawa, ON, Canada K1H 8L6; ^4^Division of Maternal-Fetal Medicine and Cellular and Molecular Medicine, Departments of Obstetrics and Gynecology, Faculty of Medicine, University of Ottawa, 501 Smyth Road, Ottawa, ON, Canada K1H 8L6; ^5^Department of Pediatrics, Faculty of Medicine, University of Ottawa, 501 Smyth Road, Ottawa, ON, Canada K1H 8L6

## Abstract

The quality of the intrauterine environment influences maternal-fetal health and also offspring predisposition to obesity and cardiometabolic disease later in life. Several determinants, including but not limited to pregravid obesity and excessive gestational weight gain, alter the developmental milieu, fetal growth, and child obesity risk. However, the role of sleep and its relationship to healthy pregnancy is not fully established. Given the host of psychosocial and physiological complications associated with childhood obesity, targeting the gestational period is purported to be an opportune time for preventive intervention. Many longitudinal studies suggest that short sleep duration is a risk factor for the development of impaired glycemia and obesity. However, there is a dearth of information concerning the role of sleep hygiene and its role in a healthy pregnancy. Reports note disrupted and poorer quality of sleep during gestation and highlight an association between reduced sleep and risk of gestational diabetes mellitus. Given the lack of well-designed human trials assessing the value of sleep and healthy pregnancy outcomes, this review summarizes current evidence which suggests that incorporating sleep recommendations and utilizing time management strategies that encourage a healthful night 's sleep may improve the health of the mom and the baby.

## 1. Introduction

It is widely accepted that the quality of the intrauterine environment has potential to not only influence prenatal health of the mother and developing child but may also increase offspring predisposition to obesity and cardiometabolic pathology later in life [1]. Several determinants, including but not limited to pregravid obesity and excessive gestational weight gain (GWG), can influence the developmental milieu. For instance, exceeding the 2009 Institute of Medicine upper limit of recommended gain is an independent predictor of large for gestational age neonates [2]. Moreover, observational studies show that offsprings born large are more likely to develop insulin resistance *in utero *[3] and have increased body fat percentage [4] and a 2-fold greater risk of becoming obese later in life [5] compared to those born at an appropriate birth weight. Recently, Laitinen et al. [6] demonstrated, using a prospective cohort, that excessive GWG (≥7 kg) in the first half of gestation (20 weeks) was independently associated with overweight/obesity and abdominal obesity in a 16- year- old offspring (OR 1.46 and OR 1.37, resp.). Thus, attenuating maternal overnutrition and optimizing fetal growth trajectory in the early years may decrease risk of pediatric overweight through a modifiable risk factor (i.e., GWG) during pregnancy. Given the host of psychosocial and physiological complications associated with childhood obesity, targeting the gestational period is considered an opportune time for preventive intervention as pregnancy is described as a critical period of body weight regulation for both mom and baby where lifestyle interventions are encouraged [7].

Currently, strategies that intervene during gestation primarily focus on maternal physical activity and/or improving dietary practices as a way to attenuate excessive GWG and optimize maternal-fetal outcomes [8, 9]. Although recent meta-analyses evaluating the efficacy of prenatal lifestyle interventions (i.e., nutrition or physical activity) targeting GWG management during pregnancy have shown promise [10, 11], incorporating recommendations and strategies designed to improve sleep hygiene may be another tool in our armamentarium that health care providers can use to optimize maternal-fetal health. Additionally, using a mixed-methods approach Hall et al. [12] noted that sleep was an outcome measure evaluated in a Canadian research study that mothers preferred to receive information about during pregnancy so they could benefit from reassurance that their experiences were shared among other pregnant women. In addition, these women intended to share the results they received within their social networks improving knowledge translation of the research findings to nonstudy participants.

To our knowledge, there is a dearth of information concerning the role of adequate sleep hygiene during pregnancy and how it relates to maternal-fetal outcomes. Although Chang et al. [13] noted that prenatal sleep deprivation was associated with longer labour, increased labour pain perception and discomfort, higher caesarean rates, preterm births, and postpartum depression, the reviewed studies were heterogeneous, lacked methodological rigor, prospective designs, and *a priori *hypotheses, which prevent firm conclusions from being made. Furthermore, no study to date has extensively examined the mechanisms by which short sleep impairs glycemia as related to gestational diabetes mellitus (GDM), a prevalent pregnancy-related comorbidity. Accordingly, the objective of this mininarrative review is to summarize the available evidence with respect to sleep behaviour and metabolic regulation of glycemic control, provide preliminary support for the value of adequate sleep for a healthy pregnancy, and highlight patient monitoring tools that may be useful to the care provider in clinical practice.

## 2. Pregnancy Adversely Impacts Sleep

It is well established that sleep is altered during human pregnancy [14] and trimester-specific changes in sleep architecture have been noted in animal models [15]. Furthermore, disrupted and/or poorer quality sleep during human gestation has been reported [16]. These effects have partially been attributed to rising hormone levels, predominately progesterone, and its soporific effects [17]. In addition, fetal organogenesis increases the metabolic requirements of pregnancy which may place burden on maternal energy stores. Thus, increased sleep time may be a potential mechanism to conserve energy [17]. On the other hand, as pregnancy progresses, sleep is disrupted for reasons including nocturia resulting from bladder compression due to increased uterine size, heartburn, and increased fetal movements. Sleep may also become fragmented as a result of sleep-disordered breathing due to hormonal fluctuations and/or overweight and obesity where excess weight further compresses the thorax and hinders respiratory capacity leading to altered respiration [17]. Moreover, pregnancy-related discomfort, joint pain, cramping, nausea, and an enlarged abdomen may limit sleep positions and impede sleep quality and duration. Nonetheless, there is little doubt that sleep quality and duration are significantly impacted in most pregnancies by the associated mechanical and physiological changes.

## 3. Glucose Intolerance and Weight Gain during Pregnancy

Greater than 50% of women in their childbearing years enter pregnancy overweight or obese [18] and more commonly present with impaired glucose tolerance when compared to those with normal body mass index [19]. Although maternal insulin resistance naturally occurs during pregnancy to facilitate fetal substrate delivery, short sleep duration has been cited as an independent predictor of diabetes in women [20], which may exacerbate glucose intolerance and expose the developing fetus to excessive quantities of hormones, growth factors, and nutrients. Supportive evidence is provided by observing pregnancies complicated by GDM where macrosomic infants are more common and present with increased body fat percentage [3, 21–23]. When considering the regulation of energy balance during gestation it is important to highlight that the caloric requirements of pregnancy are modest such that for normal weight and overweight women only a small caloric increase is needed during the 2nd and 3rd trimester of pregnancy equivalent to about 300 kcal/day over nonpregnant values [24]. However, many pregnant women gain weight in excess of the Institute of Medicine recommendations [2] which indicate a state of positive energy balance throughout gestation resulting from overconsumption and/or lack of energy expenditure. Understanding factors regulating energy balance during pregnancy may help optimize GWG, glycemic control, and healthy pregnancy outcomes for mom and baby.

## 4. Sleep and Glycemic Control

A combination of factors may lead to fragmented short sleep and altered glucose metabolism during pregnancy. While a lack of scientific evidence is available with respect to the effects of reduced sleep on glucose metabolism and weight gain during pregnancy, emerging evidence from the nonpregnant population suggests that insufficient sleep (short sleep duration and/or poor sleep quality) is an important predictor of impaired glycemic control, weight gain, less-than-optimal weight loss during weight-reducing interventions, and overweight and obesity incidence [25]. Furthermore, in a recent meta-analysis of observational studies, Ju and Choi [26] concluded that both short (<5-6 hours) and long (>8–10 hours) sleep durations increased risk of metabolic syndrome in 18–50- year- old adults and suggested that sleep habits are amenable to behavioural intervention. Nevertheless, in the absence of human trials examining the effects of shortened sleep on maternal-fetal outcomes, work from animal models suggests that environmental perturbation of the circadian clock by shortening or extending the natural light-dark cycles in the mouse is explicitly linked to adverse pregnancy outcomes [27]. This may thereby initiate altered behavioural responses to dyssynchronous circadian processes in early life that alter homeostatic regulation of this endogenous mechanism during growth and development.

However, the clinical utility of improved sleep hygiene during pregnancy stems from studies which note a strong association between reduced sleep and risk of GDM [28]. For instance, observational investigations suggest that short sleep duration (i.e., <7 hours per night) compromises glycemic control in patients at risk for GDM [29, 30]. In a pilot study, Qiu et al. [30] noted increased GDM risk in 1290 patients who reported short sleep (≤4 hours per night) compared with those who slept ≥9 hours per night. Interestingly, overweight women who reported short sleep or snoring had the greatest GDM risk. Similarly, Facco et al. [29] noted frequent snoring and short sleep duration to be associated with elevated oral glucose tolerance test (OGTT) values in 189 pregnant women. Similarly, Tsai and colleagues [31] suggested that interventions designed to increase sleep duration and decrease depressive symptoms have the potential to prevent, ameliorate, or reduce fatigue in pregnant women. They noted that depressive symptoms during pregnancy may share some physiological and behavioural tendencies with fatigue and/or sleep disturbance which may complicate the evaluation of an intervention effect. Furthermore, recent work from Izci Balserak et al. [32] noted that sleep-disordered breathing symptoms and total nap duration were associated with hyperglycemia, but sleep duration was not. Yet whether women with sleep-disordered breathing and/or daytime napping are at risk for gestational diabetes is unknown. Using the Epworth Sleepiness Scale (ESS) which is a measure of daytime fatigue and surrogate marker of poor nocturnal sleep, Bourjeily et al. [33] described a significant association between sleep and GDM in patients with a score >16 compared to those with an ESS of ≤16 which persisted after multiple adjustments (aOR, 6.82; 95% CI, 1.19–39.27). Finally, Reutrakul et al. [28] noted that each hour of reduced sleep time was associated with a 4% increase in glucose levels following a 50-gram OGTT during the second trimester. Accordingly, these studies suggest that impaired sleep quality or duration and daytime sleepiness impair regulation of glucose homeostasis in the pregnant population; however, the underlying mechanism remains to be established.

Pro- and anti-inflammatory cytokines are thought to remain in balance during the gestational period and play a significant role during pregnancy. However, when patients are sleep deprived an increased inflammatory response is observed in both the pregnant and nonpregnant states suggesting a potential mechanism implicated in short sleep duration and impaired glycemic control during pregnancy [34, 35]. For example, TNF-*α* levels are found to be increased in pregnant women who subjectively reported disrupted sleep [35]. This observation is of clinical relevance as an altered proinflammatory *milieu* may be implicated in dysregulated insulin signalling and thus impaired insulin sensitivity suggesting another way in which short sleep can impair glycemic control [17, 36]. Consequently, O'Keeffe and St-Onge [17] suggested that because most pregnancies, unaffected by metabolic disorders such as GDM, present with a mild degree of insulin resistance (as expected) [37], disordered sleep hygiene via reduced quality or duration of sleep may further exacerbate GDM risk. This may be of particular interest to mothers with overweight or obesity and their neonates [21, 22] as these phenotypes have increased risk for the development of insulin resistance and may be considered vulnerable clinical populations. Furthermore, as the incidence of maternal obesity in women of reproductive age rises so too does the clinical presentation obstructive sleep apnea during pregnancy; yet screening and treatment are not routine [17], highlighting the potential value of sleep in the prenatal conversation with the maternity care provider (e.g., midwife, general practitioner, and obstetrician). Therefore, improved lifestyle management with a focus on sleep hygiene may be another potential tool that health care providers can use in an attempt to help their patients attain a healthful pregnancy.

## 5. Sleep and the Regulation of Energy Balance

While it is unclear if short sleep decreases total energy expenditure, it is well-established that short sleep duration results in increased caloric intake (as reviewed by Chaput and Tremblay) [25]. In addition to short sleep our modern sedentary activities of daily living and ubiquitous exposure to an obesogenic environment promote overconsumption of food further increasing risk of excessive weight gain and adipose-related pathology [38]. In a randomized cross-over study Nedeltcheva et al. [39] described how sleep quantity contributes to the maintenance of fat-free body mass when energy intake is decreased. Here, partial sleep restriction decreased the proportion of weight lost as fat and substantially increased fat-free mass loss by 60%. Although it is not our intent to recommend dieting or weight loss during pregnancy, their findings suggest that lack of sufficient sleep may compromise efforts to achieve healthy weights during gestation.

Furthermore, in a recent clinical update on energy homeostasis and insufficient sleep, Penev [40] thoroughly described how short sleep promotes a series of neuroendocrine, metabolic, and behavioural adaptations that drive increased food intake and promote energy conservation. From an evolutionary perspective these adaptations offer tremendous benefit in times of nutrient deprivation and provide a protective mechanism that attempts to offset the metabolic demands of extended wakefulness. Currently, however, a metabolic mismatch occurs as a result of our modern obesogenic environment that facilitates overconsumption of readily accessible foods in the presence of sedentary behaviours in a population of habitually short sleepers. Consequently, this may be maladaptive and promote excessive energy storage and attenuated energy expenditure. Furthermore, it may limit the success of behavioural weight management programs as short sleepers have increased time available to eat and may be less likely to engage in regular moderate-to-vigorous physical activity. In the context of physical activity during pregnancy, adequate sleep may facilitate adherence to the joint clinical practice guideline for exercise in pregnancy and the postpartum period put forward by the Society of Obstetricians and Gynaecologists of Canada and the Canadian Society for Exercise Physiology (SOGC-CSEP) [41]. Thus, routine engagement in a healthy active lifestyle during pregnancy may help optimize maternal-fetal outcomes [42]. Collectively, the aforementioned observations suggest that improved sleep hygiene may help women achieve a healthy pregnancy with optimal weight gain and insulin sensitivity while balancing physical activity energy expenditure with energy intake.

## 6. A Good Night's Sleep to Improve Pregnancy Outcomes?

It is well established that sleep quality declines with advancing gestation [43] and early GWG is a risk factor for GDM and neonatal macrosomia [44], both risk factors for downstream child obesity in the offspring [7]. The high prevalence of pregnancy-related sleep complaints and the limited number of efficacious pharmacological treatments indicate that behavioural management of sleep hygiene is essential in early gestation when sleep quality is optimal. Thus encouraging sleep early in pregnancy and/or behaviour which facilitate improved sleep (e.g., regular physical activity participation) may help attenuate pregnancy-associated deterioration in sleep hygiene and improve maternal-fetal outcomes [42]. While we are unaware of studies that have explored regular physical activity participation and sleep quality during pregnancy, a recent study by Kjeldsen et al. reported that aerobic exercise for 13 weeks improved sleep duration and improved sleep quality in sedentary, overweight men [45]. However, future studies with larger sample size in a pregnant population warrant investigation to validate this hypothesis.

Thus, while some intervention studies demonstrate efficacious GWG management with physical activity and nutrition intervention, the effectiveness of preventive therapy in real-life situations remains to be shown. This suggests that pregnant women may find it difficult to adhere to the behaviour necessary to promote health benefits including physical activity, proper dietary intake, and sleep hygiene. Although we appreciate the need for multifactorial approaches to optimize body composition and promote long-term maintenance of healthy behaviour (e.g., physical activity) and body weight following weight loss in adults, GWG management in women adds another layer of complexity given the many physiological and anatomical changes unique to childbearing. Despite these challenges, there is clinical value to maintaining optimal energy balance that satisfies maternal-fetal needs for growth and development while minimizing risk of obesity, postpartum weight retention, and adipose associated pathologies [7]. While we are not recommending restrictive dieting or negative energy balance for pregnant women, a more precise matching of caloric expenditure with intake through behavioural modification that incorporates physical activity, healthful nutrition, and adequate sleep may help to optimize glycemic control and adherence to GWG upper limits for each pregravid BMI category. Thus, it is important to recognize the multiple behaviour that must be addressed during pregnancy including sleep, physical activity, and healthful nutrition as comprehensive lifestyle management is likely to improve adherence to GWG recommendations and in turn optimize maternal-fetal outcomes [7].

Of great interest are the many longitudinal studies suggesting that short sleep duration is a risk factor for the development of obesity [46, 47]. Despite a lack of definitive human studies that preclude causal conclusions from being made, the fact that short sleep does not seem to drastically affect total daily energy expenditure in humans has put forward the notion that increased energy intake is the mediating factor driving this relationship [48]. Specifically, less sleep equals higher energy intake which may in turn lead to obesity if chronic sleep deprivation and commensurate overconsumption of food persists. Thus, if lack of sleep promotes positive energy balance, weight, gain and central adiposity through increased calorie consumption [48] and restoration of adequate sleep in identified short sleepers protects against further weight gain [49], it seems reasonable to hypothesize that adequate sleep may be of health benefit during pregnancy. It is theorized that a consequence of better sleep may be greater engagement in healthy lifestyles, further balance energy consumption with energy expenditure during pregnancy, and yield optimal glycemic control and potentially GWG at term. Conversely, weak associations of physical activity with sleep quality and duration in late pregnancy exist suggesting a need for further study before physical activity recommendations can be made to improve sleep hygiene during pregnancy for all women [50].

Moving beyond pregnancy to the postpartum period, optimizing sleep behaviour may improve attempts to limit postpartum weight retention (PPWR) and maintain optimal glycemic control through a similar mechanism. For instance, Thomson et al. [51] studied the effects of sleep hygiene on weight loss in women enrolled in a randomized clinical trial and by using the Pittsburgh Sleep Quality Index (PSQI) noted that both improved sleep quality and greater sleep quantity (>7 h/night) contributed to weight loss success and promoted weight control in overweight and obese adult women. Similarly, Nedeltcheva et al. [39] noted that lack of sleep compromises efficacy of dietary interventions designed for weight loss and metabolic risk reduction. Interestingly, disappointing weight loss among shift workers after gastric bypass surgery has also been reported [52], suggesting that altered sleep cycles impair metabolic control of energy balance. Although this is merely speculative at the current time given the lack of literature in humans concerning sleep, GWG, and PPWR, incorporating sleep recommendations and utilizing time management strategies that encourage a healthful night's sleep may improve efforts to achieve a healthy pregnancy early in gestation through to the postpartum period.

## 7. Future Research

With respect to human pregnancy and sleep behaviour, much work needs to be done to determine if proper sleep hygiene improves maternal-fetal health. Currently, whether the mechanisms mediating the relationship between short sleep duration and glycemic control differ between pregnant and nonpregnant women is not known. Additionally, as highlighted by O'Keeffe and St-Onge [17], further research is needed to determine if sleep interventions that promote increased duration or improved quality of nocturnal sleep and/or those which promote daytime naps would yield optimal glycemic control and counteract the adverse effects of short sleep. It is our contention that a randomized controlled trial evaluating the effects of a sleep extension intervention on maternal-fetal outcomes is an area ripe for exploration and offers great clinical potential. Similarly, a nested case-control study from an ongoing prospective pregnancy cohort may help inform clinical decisions with respect to sleep behaviour and maternal-fetal outcomes. Lastly, when considering measurement of outcomes it is important to consider the work of Wilson et al. [53] who compared self-reported sleep latency and total sleep time to objective measures using polysomnography (PSG) during pregnancy. They noted that the group of women in the third trimester slightly overestimated their total sleep time while those in the first trimester and nonpregnant women underestimated total sleep time when compared with objective measures. Similarly, recent evidence from Herring et al. noted that pregnant women underreport sleep time when compared to direct measurement [54]. However, the logistics of implementing direct measures of sleep hygiene during pregnancy may be difficult despite providing precise outcome measures. Although directly measuring sleep patterns with PSG may be an advantageous assessment strategy for at risk individuals, the use of wrist actigraphy at home to objectively measure sleep duration, quality, and efficiency may be a valid and reliable compromise to help inform clinical decisions that improve sleep hygiene.

## 8. Practicalities of Sleep Assessment

The Pittsburgh Sleep Quality Index (PSQI) may be one way in which health care providers can gather information about their patients' sleep quality and duration. Additionally, in a report targeted to primary care providers, Blythe et al. [55] outlined techniques for screening and diagnosing common sleep/wake disorders (e.g., obstructive sleep apnea, insomnia related to inadequate or poor sleep quality, etc.). Recently, the Canadian Obesity Network developed and validated “The 5 As of Obesity Management” for a nonpregnant population to give health practitioners five steps to better manage their patients' weight and related health issues (available at http://www.obesitynetwork.ca/) [56]. Currently, a revised 5 As for pregnancy is under development. In both sets of care provider tools sleep hygiene in the context of body weight regulation is addressed. Additionally, Foxcroft et al. [57] developed and validated a Pregnancy Symptoms Inventory (PSI) for health professionals to assess pregnancy related symptoms and their effect on functional capacity during gestation A 41-item Likert inventory that includes items which assess fatigue (i.e., tiredness), sleep, and pain, among others, was robustly developed, with good test-retest reliability, face validity, comprehension, and readability. This may provide a validated tool for assessing the impact of interventions in pregnancy. Overall, novel clinical aids designed to help physicians better manage patient weight that incorporate messaging on sleep hygiene complement existing evidence supporting a balance of other modifiable lifestyle related factors including healthful nutrition and physical activity to improve maternal-fetal health.

## 9. Concluding Remarks

Emerging evidence links insufficient sleep with adiposity gains over time, suggesting the need to inform all patients about the value of sleep hygiene as a preventive measure. These recommendations should not be limited to the nonpregnant population but extended to expecting mothers as well. Given the complex nature of body weight regulation and the poor maternal-fetal outcomes associated with impaired glycemic and excessive GWG during pregnancy, sleep may be another talking point for practitioners to assess and advise on during prenatal counselling that may help optimize glycemic control and at the very least improve metabolic health.
